# Quality and Safety Requirements for Sustainable Phage Therapy Products

**DOI:** 10.1007/s11095-014-1617-7

**Published:** 2015-01-14

**Authors:** Jean-Paul Pirnay, Bob G. Blasdel, Laurent Bretaudeau, Angus Buckling, Nina Chanishvili, Jason R. Clark, Sofia Corte-Real, Laurent Debarbieux, Alain Dublanchet, Daniel De Vos, Jérôme Gabard, Miguel Garcia, Marina Goderdzishvili, Andrzej Górski, John Hardcastle, Isabelle Huys, Elizabeth Kutter, Rob Lavigne, Maia Merabishvili, Ewa Olchawa, Kaarle J. Parikka, Olivier Patey, Flavie Pouilot, Gregory Resch, Christine Rohde, Jacques Scheres, Mikael Skurnik, Mario Vaneechoutte, Luc Van Parys, Gilbert Verbeken, Martin Zizi, Guy Van den Eede

**Affiliations:** 1 Laboratory for Molecular and Cellular Technology, Queen Astrid Military Hospital, Bruynstraat 1, 1120 Brussel, Belgium; 2 Laboratory of Gene Technology, KU Leuven, 3001 Leuven, Belgium; 3 Clean Cells SAS, Parc d’Activités Vendee Sud Loire, 85600 Bouffere, France; 4 Department of Biosciences, University of Exeter, Penryn, TR10 9EZ UK; 5 George Eliava Institute of Bacteriophages, Microbiology and Virology, 0160 Tbilisi, Georgia; 6 Novolytics Limited, Daresbury Science and Innovation Campus, Warrington, WA4 4AD UK; 7 TechnoPhage SA, Edifício Egas Moniz, 1649-028 Lisbon, Portugal; 8 Department of Microbiology, Molecular Biology of the Gene in Extremophiles Unit, Institut Pasteur, 75724 Paris, France; 9 Service de Médecine Interne, Maladies Infectieuses et Tropicales, Centre Hospitalier Intercommunal, 94195 Villeneuve Saint-Georges, France; 10 Pherecydes Pharma, 93230 Romainville, France; 11 Institute of Immunology & Experimental Therapy, Polish Academy of Sciences, 53-114 Wrocław, Poland; 12 The Medical University of Warsaw, 02-006 Warsaw, Poland; 13 Clinical Pharmacology and Pharmacotherapy, KU Leuven, 3000 Leuven, Belgium; 14 The Evergreen State College, Olympia, Washington 98505 USA; 15 Laboratory for Bacteriology Research, Faculty of Medicine, Ghent University, 9000 Ghent, Belgium; 16 Instytut Biotechnologii Surowic I Szczepionek Biomed S. A., 30-224 Kraków, Poland; 17 Belgian Defense Staff, Medical Operational Command (COMOPSMED) Quartier Reine Elizabeth, 1200 Brussel, Belgium; 18 Department of Physiology, Free University Brussels, 1090 Brussel, Belgium; 19 Department of Fundamental Microbiology, University of Lausanne, 1015 Lausanne, Switzerland; 20 Department of Microorganisms, Leibniz Institute DSMZ - German Collection of Microorganisms and Cell Cultures, 38124 Braunschweig, Germany; 21 National Institute of Public Health NIZP, 00-791 Warsaw, Poland; 22 European Centre for Disease Prevention and Control (ECDC) Management Board, 171 83 Stockholm, Sweden; 23 Haartman Institute, Department of Bacteriology and Immunology, and Research Programs Unit, Immunobiology, University of Helsinki, 00014 Helsinki, Finland; 24 Helsinki University Central Hospital Laboratory Diagnostics, Helsinki, Finland; 25 5th Element of Medical Intervention (5 EMI), 1400 Nijvel, Belgium; 26 European Commission Joint Research Centre, Scientific Policy and Stakeholder Relations, 1049 Brussel, Belgium

**Keywords:** antibiotic resistance, medicinal product, phage therapy, production, quality and safety

## Abstract

The worldwide antibiotic crisis has led to a renewed interest in phage therapy. Since time immemorial phages control bacterial populations on Earth. Potent lytic phages against bacterial pathogens can be isolated from the environment or selected from a collection in a matter of days. In addition, phages have the capacity to rapidly overcome bacterial resistances, which will inevitably emerge. To maximally exploit these advantage phages have over conventional drugs such as antibiotics, it is important that sustainable phage products are not submitted to the conventional long medicinal product development and licensing pathway. There is a need for an adapted framework, including realistic production and quality and safety requirements, that allowsa timely supplying of phage therapy products for ‘personalized therapy’ or for public health or medical emergencies. This paper enumerates all phage therapy product related quality and safety risks known to the authors, as well as the tests that can be performed to minimize these risks, only to the extent needed to protect the patients and to allow and advance responsible phage therapy and research.

Antimicrobial resistance in bacteria is an increasingly serious threat in every part of the world [1]. Without action, the world could be heading towards a post-antibiotic era in which common infections become fatal and currently routine surgeries become impossible. New initiatives to tackle the problem of antibiotic resistance are urgently needed.

One promising solution is the therapeutic use of bacteriophages – the viruses of bacteria, also known as phages – to treat bacterial infections. When discovered in the early twentieth century, phages were immediately applied in medicine (phage therapy) with variable success. After World War II, Western industry and policymakers preferred antibiotics, which at the time had obvious advantages in terms of breadth of coverage and ease of production and patentability, and phage therapy was pushed into the background. Today, phage therapy is again put forward as a potential way to address the current antibiotic crisis [2, 3].

Since time immemorial, phages have controlled bacterial populations on our planet, locked in an evolutionary arms race with their hosts (consisting of the repeated emergence of new phage infectivity and bacterial defense mutations). The capacity of bacteriophages to rapidly overcome bacterial resistance makes them suitable for flexible therapeutic applications. To maximally exploit this key advantage of phages over conventional ‘static’ drugs such as traditional small molecule-type antibiotics, it is important that sustainable phage products are not submitted to the conventional long medicinal product development and licensing pathway [4]. A key goal for the modern phage therapy community must be the development and validation of an expedited product development and licensing pathway in consultation with policymakers and competent authorities.

Georgian and Polish phage therapy centers maintain extensive therapeutic phage collections, which are regularly enriched with new phages, thus widening the total host range of the collection and adapting the collection to changing bacterial populations (with regard to host range and antibiotic resistance as well as phage resistance). Moreover, the effectiveness of phages can be readily improved by *in vitro* selection of (natural) phage mutants that exhibit an increased infectivity range. For example, it is possible to obtain potent lytic phages against problematic enteroaggregative *Escherichia coli* strains by isolation of new phages from the environment or by selection and adaptation of phages from an existing collection, and this often in a matter of days [5]. As such, phages could probably have been used to help control the O104:H4 (hybrid EAggEC STEC/VTEC pathotype) *E. coli* outbreak that caused the death of more than 50 patients in Germany in 2011. Unfortunately, authorized use of phages would not have been possible in this otherwise feasible context because under the existing medicinal product legislation such an anti-O104:H4 phage preparation would have taken years to develop, produce and register. Since phages are species and often even strain-specific, it is very likely that current O104:H4‑specific phage preparations will not be effective against future epidemic enteroaggregative *E. coli* strains. ‘Broad‑spectrum’ phage cocktails active against bacteria that are likely to cause problems in the future could be developed in advance and used as a first line treatment for acute healthcare problems (*e.g.,* foodborne disease outbreaks and bacterial bioweapon threats). However, we need to keep in mind that some of these cocktails will not always work due to the greater biodiversity outside of the laboratory and the existing resistance to specific phages. The cocktails that initially work will need to be regularly updated (*e.g.,* supplemented with new phages in response to the evolution of phage resistance and the involvement of new circulating bacterial strains). There are indications that bacterial resistance to phages, even to cocktails containing multiple potent phages, will inevitably occur [6].

Notwithstanding the Intellectual Property (IP) and regulatory hurdles, as well as the empirical evidence suggesting that stable and widely distributed phage preparations (*prêt-à-porter*) will need to be constantly updated, a few companies have picked up the gauntlet and are slowly moving along the elaborate and expensive conventional medicinal product licensing pathway. The development and marketing of phage medicinal products in the EU – including Good Manufacturing Practice (GMP) production, preclinical and Phase I, II and III clinical trials and centralized marketing authorization – is in fact technically possible (and indeed advisable for some products), providing some minor modifications and logical exemptions are made.

However, multiple discussions between experts, competent authorities and policymakers have led to an increasing awareness that *sustainable* (*sur-mesure*) phage therapy is not compatible with the conventional approaches to the development and application of medicinal products [4]. Next to the classical medicinal product pathway, which should be adjusted to support the industrial production of (first line) broad-spectrum phage cocktails or phage-derived products (*e.g.,* phage endolysins), there is a need for a specific framework (including realistic production and quality and safety requirements) that allows a timely (rapid) supplying of adapted productions of natural bacteriophages for ‘personalized therapy’. This regulatory framework could be based on the Quality by Design (QbD) concept, which is increasingly applied to the development and production of biopharmaceutical molecules [7]. The QbD approach entails designing quality into the process and the product, and this in a science- and risk-based manner. Understanding patients’ needs and determining the specific science and quality characteristics of the product that are linked to safety and efficacy are crucial components of QbD. More research is urgently needed to gather the required data with regard to the efficacy of phage therapy and to broaden our understanding of bacteria-phage coevolution in nature and in the context of human disease [8, 9]. To avoid the mistakes of the past (which lead to the current antibiotic resistance crisis), phage therapy products should not exclusively be developed and marketed as antibiotics, *i.e.,* applying current pharmacoeconomic principles. Ideally, phage therapy should be coordinated and standardized (in a first instance) by national phage therapy centers, which operate under the supervision of relevant public health authorities and in interaction with private stakeholders.

There are precedents for such a dedicated ‘non-medicinal product’ approach. In the European Union (EU), human tissues and cells that are not considered as ‘Advanced Therapy Medicinal Products (ATMPs)’ are procured, processed, tested and allocated by (or under the responsibility of) dedicated tissue establishments and are exclusively regulated by the EU Tissue and Cell Directives (EUTCDs). The EUTCDs consist of three Directives, the parent Directive (2004/23/EC), which provides the framework legislation, and two technical Directives (2006/17/EC and 2006/86/EC), which provide the detailed requirements of the EUTCD. The purpose of these Directives was to facilitate a safer and easier exchange of human tissues and cells between member states and to improve safety standards for European citizens. They set a benchmark for the standards that must be met when carrying out any activity involving tissues and cells for human application.

In view of further meetings with phage experts and representatives of the competent authorities and policymakers – coordinated by the European Commission Joint Research Centre, which acts in an advisory capacity to the Commission and its policy making directorates general –, a group of ‘phage experts’ (the authors of this paper) were asked through the intermediary of a not-for-profit organization (www.p-h-a-g-e.org) to set realistic quality and safety requirements for sustainable phage therapy products (Table I). These requirements are intended to apply to the production of phage therapy products (finished products), starting from banked characterized natural therapeutic bacteriophages (Master Seed lots), and possibly using intermediate bacteriophage products (Working Seed lots or Active Substances). They were roughly based on the EUTCD quality and safety standards for human cells and were defined by consensus among 32 phage experts (biologists, geneticists, bioengineers, quality managers, pharmacists and MDs) from 12 countries. This document enumerates all possible phage product related quality and safety risks known to the experts, as well as the tests that can be performed to minimize these risks, only to the extent needed to protect the patients and to allow and advance responsible phage therapy and research. The exact tests used and limits applied will depend on the route of administration (*e.g.,* topical or systemic) and the regulatory path the product is being used under. These requirements do not address efficacy aspects of phage therapy products.

**Table I:** Expert Consensus Quality and Safety Requirements for Sustainable Phage Therapy Products

A. Production environment			
When production activities include the processing of intermediate, bulk or finished phage products exposed to the environment, this must take place in an environment with specified air quality and cleanliness in order to minimize the risk of contamination. The effectiveness of these measures must be validated and monitored. Where intermediate, bulk or finished products are exposed to the environment during processing, without a subsequent microbial inactivation process, an *air quality* with particle counts and microbial colony counts equivalent to those of Grade A as defined in the current European Guide to Good Manufacturing Practice (GMP), Annex 1 and Directive 2003/94/EC is required with a background environment at least equivalent to GMP Grade D in terms of particles and microbial counts. The biosafety level (BSL) is determined by the host bacteria used in the production processes (*e.g.,* BSL-2 for *Pseudomonas aeruginosa*).			
B. Production processes, equipment and materials			
All equipment and material must be designed and maintained to suit its intended purpose and must minimize any hazard to recipients and staff. All critical equipment and technical devices must be identified and validated, regularly inspected and preventively maintained in accordance with the manufacturers’ instructions. Where equipment or materials affect critical processing or storage parameters (*e.g.,* temperature, pressure, particle counts, microbial contamination levels), they must be identified and must be the subject of appropriate monitoring, alerts, alarms and corrective action, as required, to detect malfunctions and defects and to ensure that the critical parameters are maintained within acceptable limits at all times. All equipment with a critical measuring function must be calibrated against a traceable standard if available. Maintenance, servicing, cleaning, disinfection and sanitation of all critical equipment must be performed regularly and recorded accordingly.			
Production processes must be described in detail (equipment, materials, culture media, additives, culture conditions, purification steps,..) in standard operating procedures (SOPs) and must be validated (procedures published in relevant peer-reviewed journals could be considered ‘validated’).			
SOPs must detail the specifications for all critical materials and reagents. In particular, specifications for culture media, additives (*e.g.,* solutions) and packaging materials must be defined. Critical reagents and materials must meet documented requirements and specifications and when applicable the requirements of Council Directive 93/42/EEC of 14 June 1993 concerning medical devices and Directive 98/79/EC of the European Parliament and of the Council of 27 October 1998 on *in vitro* diagnostic medical devices. If possible, animal component free culture media and additives should be used (the Note for Guidance on Minimizing the Risk of Transmitting Animal Spongiform Encephalopathy Agents *via* Human and Veterinary Medicinal Products (EMEA/410/01) in its current version is to be applied). If animal–product free media are not used, Transmitting Animal Spongiform Encephalopathy (TSE)-free certification should be obtained for all components containing products of animal origin.			
Analytical methods can be validated according to: a) EMEA/CHMP/EWP/192217/2009 “Guideline on bioanalytical method validation” or b) CPMP/ICH /381/95 “ICH Topic Q 2 (R1) Validation of Analytical Procedures: Text and Methodology”.			
Bacteria and phage bank systems need to be set up. These bank systems typically consist of Master seed lots and Working seed lots. The generation and characterization of the banks should be performed in accordance with principles of CPMP/ICH guideline Q5D. The banked phages and bacteria should be characterized for relevant phenotypic and genotypic markers so that the identity, viability (activity for phages), and purity of organisms used for the production are ensured. Biological Resource Centers [10] could function as repositories for bacteriophage Master Seeds and host bacteria.			
C. Quality Assurance and Quality Control (QA/QC) specifications			
Products/characteristics	Control test	Limits of acceptance	Recommended test procedures
C.1. Host bacteria used in production (stock suspensions)			
The bacterial hosts used in the production process – with the exception of selection, adaptation and efficiency of plating (EOP) and host range determination – should be as safe (or least pathogenic) as feasible.			
Origin	Document pedigree/history/pathogenicity level	Known origin	Screening of scientific literature, lab books, consignment letters,..
Identification	Identification at the species and strain levels	Matching species and strain identification	• State of the art clinical microbiology techniques
			• Highly discriminating (molecular/genomic) typing techniques (*e.g.,* MLST, AFLP, PFGE, Rep-PCR,..)
Most often it will not be possible to find or quickly generate a suitable host bacterium that is free of prophages or phage-like elements, but one should nevertheless strive to use non-lysogenic strains, containing as few phages or other phage-like elements of genetic exchange [11, 12] as possible	• Induction of phages	As few spontaneously produced (or by induction) temperate phages, complete prophage sequences or phage-like elements as possible^a^	• *In vitro* induction methods (Mitomycine C [13] or UV induction)
	• Host genome screening for phage or phage-like elements		• State of the art DNA sequencing and analysis (bioinformatics) procedures
Avoid mutator strains as host bacteria	Screen for mutator strains in case of doubt	No mutator strain	State of the art tests (*e.g.,* fosfomycin and rifampicin Disk Diffusion Tests) [14]
Validated preservation/storage (cryopreservation, freeze-drying,..)	Monitor storage conditions (*e.g.,* temperature)	Variable, depending on the preservation method	Variable (*e.g.,* temperature probes, temperature indicator labels,..)
C.2. Bacteriophages (Master Seed lots)			
Origin	Document bacteriophage pedigree/history (*e.g.,* isolation source)	• Known origin	Screening of scientific literature, lab books, consignment letters,…
		• Natural or naturally evolved bacteriophages	
Identification	• Identification at the family (subfamily), genus and species and strain level	Matching identification, morphology and biology	• State of the art DNA or RNA sequencing and analysis procedures
	• Morphology and biology		• Highly discriminating genotyping techniques (*e.g.,* AFLP, fRFLP [15])^b^
			• State of the art classification according to the International Committee on Taxonomy of Viruses (ICTV)
			• State of the art electron microscopy (optional)^c^
			• One step growth curve [16]
Not containing potentially damaging genetic determinants (*e.g.,* conferring toxicity, virulence, lysogeny or antibiotic resistance)	Genome analysis for known potentially damaging genetic determinants	Absence of potentially damaging genetic determinants^d^	• State of the art DNA or RNA sequencing and genome analysis (bioinformatics) procedures
Non-transducing (optional) [17]	Screen for ‘general transduction’	Does not pack random host DNA in a portion of progeny phage particles^e^	Transduction assay [18]
*In vitro* efficacy	Determination of host range on a panel of target species (reference) strains	Broad host range (if possible)	• Titration of bacteriophages against target bacteria according to the soft-agar overlay method [19]
		Variable threshold according to species (*e.g.,*	
		>75% for *Staphylococcus aureus*)	• Spot test [16]
	Stability of lysis (optional)^f^	Stable lysis in broth culture for 24–48 h	Appelmans method [20]
	Efficiency of plating (EOP) under conditions similar to eventual clinical application (optional)	Threshold EOP value	EOP determination [19]
	Determination of frequency of emergence of phage-resistant bacteria	Low frequency of emergence of resistance	Method described by Adams [19]
Improvement / adaptation / ‘training’ (if warranted)	Optimization of host range	Broadened and stable host range	• Titration of bacteriophages against target bacteria according to the soft-agar overlay method [16]
			• Spot test [16]
Validated preservation/storage (cryopreservation, freeze-drying,..)	Monitor storage conditions (*e.g.,* temperature)	Variable, depending on the preservation method	Variable (*e.g.,* temperature probes, temperature indicator labels,..)
C.3. Bacteriophages (Working Seed lots/Active Substances)			
Quantitative determination of active substance (bacteriophages)	Bacteriophage titration	Variable. Typically log(8) – log(10) plaque forming units (pfu)/ml	Soft-agar overlay method [19]
Identification of active substance	Genomic fingerprinting	Matching genomic fingerprint (max. deviation depends on method)	State of the art genotyping techniques (*e.g.,* AFLP, fRFLP [15])
Microbial contamination	Sterility (when there is no sense of urgency)^g^	Sterile (absence of micro-organisms)	Membrane filtration method based on the European Pharmacopoeia (EP)
	Absence of pathogens (when there is a sense of urgency)	Aseptic (absence of pathogens)	State of the art clinical microbiology methods
Toxicity	Bacterial endotoxin or lipopolysaccharides (LPS) quantification [21]	Depends on posology and method and route of administration. The maximum level for intravenous applications for pharmaceutical and biological products is set to 5 endotoxin units per kg of body weight per hour (EP).	Limulus Amebocyte Lysate (LAL) assay according to the EP (*e.g.,* kinetic-QCL method)
Bacterial DNA contamination^h^	Screen for (potentially damaging) host bacterial DNA	Absence of potentially damaging genetic determinants that are known to be present in the host bacterium	Methods for the quantification of bacterial DNA in general (*e.g.,* PicoGreen) or for the quantification of known DNA sequences (*e.g.,* qPCR)^i^
Acidity or basicity of aqueous solution	pH measurement	Variable (typically 6,5–7,5)	pH test (EP method)
Purity	Clarity of phage solution	Absence of visible particles	EP method, CPMP-ICH guideline
Validated preservation/storage (cooling, cryopreservation, freeze-drying,..)	Monitor/record/demonstrate storage conditions (temperature,..)	Variable (*e.g.,* 2–8°C)	Variable (*e.g.,* temperature probes, temperature indicator labels,..)
C.4. Finished products			
Bulk products may be diluted (typically to log(5)–log(7) pfu/ml), combined or added to a carrier (hydrogel, ointment, cream, bandage,..) prior to clinical use. Dilution solutions, carriers and packaging materials must meet documented requirements and specifications and when applicable the requirements of Council Directive 93/42/EEC of 14 June 1993 concerning medical devices. Carriers must be chosen that allow the required phage activity during the intended application period (stability).			
The following information must be provided either on the label or in accompanying documentation: (a) description (definition) and, if relevant, dimensions of the bacteriophage product; (b) date of production of the bacteriophage product (c) storage recommendations; (d) instructions for opening the container, package, and any required manipulation/reconstitution; (e) expiration dates (incl. after opening/manipulation); (f) instructions for reporting serious adverse reactions and/or events; (g) presence of potentially harmful residues (*e.g.,* antibiotics, ethylene oxide); (h) contraindications; (e) how to dispose of unused (expired) bacteriophage products.			
Validated storage (cold storage,..)	Monitor/record/demonstrate storage conditions (temperature,..)	Variable (*e.g.,* 2–8°C)	Variable (*e.g.,* temperature probes, temperature indicator labels,..)
D. Shelf life of phage stock suspensions, working solutions and finished products (at recommended storage conditions)			
Stability	• Periodic quantitative determination of the active substances (bacteriophages) or breakdown products	The shelf life is the time period during which the product remains sterile and the activity and pH remain within specified limit thresholds	• Soft-agar overlay method [15]
	• Periodic determination of sterility		• CPMP-ICH guideline, Q5C, Q1A
	• Periodic pH measurements		• Membrane filtration method (EP method)
			• pH test (EP method)
E. Surveillance			
The clinical use of phage therapy products must be surveyed and reported, including possible adverse events and reactions associated with the use of phage therapy products. A centralized (publicly available) reporting system is warranted.			

^a^Today it may be impossible to successfully cure some host strains that are indispensable for the production of some therapeutically interesting phages. In addition, in some cases it might be necessary to use phages that were isolated from the patient’s bacteria and that are not able to replicate in known host strains devoid of prophages. However, since that sort of phage preparations are only designed to be used in that given patient, any remaining traces of DNA from that host bacterium would be orders of magnitude less than the amount already present in the patient from whom that bacterium was isolated for this purpose

^b^This genetic fingerprint can be used to timely identify bacteriophages and confirm their presence in Working Seed lots and in finished products, without having to re-perform full genome sequencing. It is however expected that fast, low-cost and accurate full genome sequencing and analysis (of bacteriophages) will replace routine microbial genotyping techniques in the near future

^c^In some cases (*e.g.,* novel bacteriophages with no homology in databases), electron microscopy could provide important information and could thus be warranted

^d^In general, it is recommended to only use lytic phages (and no temperate phages) in phage therapy. Lytic phages are more potent killers of host bacteria, making them more effective in therapy than temperate phages. Following lysogenic induction, temperate phages may transfer fragments of host bacterial DNA into non-targeted bacteria (possibly belonging to other species). This phenomenon is called transduction or phage-mediated horizontal gene transfer (HGT). If these DNA fragments contain toxin-encoding or antibiotic resistance-mediating genes, temperate phages could thus produce new pathogenic strains. However, in the future, the dogma that the use, in treatment, of temperate phages is impossible or undesirable because of the danger of HGT might be abandoned in certain circumstances (science- and risk-based decision, taking into consideration the patients’ needs). In certain bacterial species, the number of strictly virulent phages is small and it might not be possible to isolate adequate new virulent phages in due time. Phage mediated HGT is abundant and virtually ubiquitous in bacterial populations and the additional and immediate danger to the patient related to the use of temperate phages in the course of phage therapy (days) is bound to be limited. Moreover, if a temperate phage acts as a lytic phage in relation to a particular pathogen, the probability of HGT might not be higher than for inherent genetic virulent phages [22]. In the future, temperate phages might specifically be used in therapy*, e.g.,* to introduce, by lysogenization, genes conferring sensitivity to antimicrobials [23] or to inhibit virulence traits [24]. Finally, antibiotic stress was also shown to induce genetic transformability in human pathogens [25]

^e^Today, it is not feasible to exclude the possibility of low levels of generalized transduction by therapeutic phages into any of the infecting and commensal bacteria present in or on the patient. The use in phage therapy of phages that mediate some random general transduction might be considered in certain circumstances (science- and risk-based decision, taking into consideration the patients’ needs)

^f^In some cases, phages that produce stable lysis will not be found in a timely fashion. Phages that induce relatively fast *in vitro* bacterial resistance might then be considered

^g^In some cases, sterility may not be required (*e.g.,* ‘non-sterile for topical application’)

^h^Working Seed lots can be contaminated with low levels of DNA derived from the host bacteria used in production. Potentially damaging genetic determinants (*e.g.,* conferring toxicity, virulence or antibiotic resistance) might then be transferred (through transformation) to bacteria present in or on the patient, which could potentially make them (more) pathogenic. While this would be expected to occur at a level well below exchanges already going on within the patient’s body involving their own pathogenic bacteria and phages already resident it makes sense to select hosts that are as devoid of pathogenicity factors as reasonably possible for growing therapeutic phage and treating the phage with DNase in the course of their purification to destroy such contaminants. If no non-pathogenic bacterial strain is available for growing the phage, constructing a ‘defanged’ host strain, with all pathogenicity determinants deleted, could be envisaged as the best main step in avoiding this issue. Note that the use of non-pathogenic host bacterial strains also reduces the potential hazard to the personnel involved in the production of therapeutic phages

^i^A threshold level should be determined. Note that some DNA quantification methods might also pick up phage DNA

Should bacteriophages be used for a public health or medical emergency and no adequate finished products, Master Seed lots or Working Seed lots are available, then less stringent requirements could be considered, pending compliance (as quick as possible) to the quality and safety requirements.
